# Melatonin maintains the function of the blood redox system at combined ethanol-induced toxicity and subclinical inflammation in mice

**DOI:** 10.1007/s11325-020-02191-1

**Published:** 2020-09-23

**Authors:** Natalia Kurhaluk, Halyna Tkachenko, Oleksandr Lukash, Pawel J. Winklewski, Magdalena Wszedybyl-Winklewska

**Affiliations:** 1 Department of Biology, Institute of Biology and Earth Sciences, Pomeranian University in Słupsk, Arciszewski Str., 22b, 76-200 Słupsk, Poland; 2 Department of Ecology and Nature Protection, National State University of Chernihiv, Chernihiv, Ukraine; 3 Department of Human Physiology, Medical University of Gdańsk, Gdańsk, Poland; 4 Department of Clinical Anatomy and Physiology, Pomeranian University in Słupsk, Słupsk, Poland

**Keywords:** Melatonin, Ethanol, LPS, Oxidative stress, Blood cells

## Abstract

**Background:**

The goal of this study was to assess the effect of melatonin on blood redox systems in mice simultaneously exposed to ethanol and low-dose lipopolysaccharide (LPS).

**Methods:**

Oxidative stress parameters were assessed in eight groups: untreated control, melatonin (10 mg kg^−1^, 10 days), LPS (injected once intraperitoneally at a dose of 150 μg per mouse), LPS with previous melatonin treatment, acute ethanol-induced stress (AES, 0.75 g kg^−1^ per day, 10 days), AES with previous melatonin treatment, LPS- and AES-induced toxicity, and melatonin treatment.

**Results:**

Both ethanol and LPS induced oxidative stress. The combination of these two factors was even more toxic to the organism. Melatonin stabilized erythrocyte membranes and decreased the high level of free radical oxidation at the initial and final stages. Furthermore, melatonin limited protein damage through maintenance in the functional ability of the blood redox system to counteract pathological conditions.

**Conclusions:**

Melatonin limited the negative effects associated with alcohol consumption and low-intensity inflammation.

## Introduction

Alcohol abuse is a major risk factor for several diseases such as liver cirrhosis, pancreatitis, cancers, and cardiovascular, neurological, or psychiatric disorders. Intestinal Gram-negative bacterial overgrowth and subsequent hyper-permeability and leakage of lipopolysaccharides (LPS) into the blood are increasingly recognized as a mechanism promoting alcohol-related pathologies [1]. LPS, the main component of the Gram-negative bacterial membrane, triggers a low-grade inflammatory response and causes oxidative stress [2, 3]. Low-intensity inflammation, in turn, has been implicated in mood and cognitive modifications, which can lead to an increased risk of alcohol dependence, thus creating a vicious cycle [4].

The use of melatonin (Mel) in patients with alcohol-induced dependencies as a substitution therapy for insomnia is dictated by the fact that there are data on the fact of reduction and even complete cessation of Mel production by epiphysis in chronic alcohol intoxication. In this case, it seems clinically justified to use exogenous Mel, in particular, in alcohol-dependent patients primarily for normalization of sleep and recovery of the daily sleep-waking cycle in the period of alcohol abstinence. The most sensitive symptoms to Mel therapy were sleep time, duration of sleep, and quality of sleep, and the least sensitive were the number of night awakenings, number of dreams, and quality of morning awakening. Reduced sleep time is because Mel shifts the sleep phase to an earlier time, as the drug has both sleeping pills and chronobiotic effect, i.e., regulates biological rhythms [5].

In a previous study, we have shown that alcohol-induced oxidative stress can be diminished by melatonin supplementation [6]. In particular, melatonin exerts protective effects in ethanol-intoxicated animals by restoring white blood cell count, diminishing glycated hemoglobin, and reducing exaggerated oxidative stress in the liver, kidney, and muscles. Moreover, we have also demonstrated that low-intensity inflammation can be ameliorated by melatonin treatment [7, 8]. In these studies, melatonin diminished oxidative stress in the liver and kidney, and restored white blood cell count, ameliorated erythrocyte membrane damage, and decreased overall oxidative processes in plasma of animals exposed to low dose of LPS.

Red blood cells (RBC) are well equipped, with multiple non-enzymatic and enzymatic antioxidant systems, to maintain hemoglobin (Hb) in a reduced, oxygen-binding form; diminish oxidative modifications to membrane lipids, structural proteins, channels, and metabolic enzymes; and, consequently, maintain erythrocyte function and integrity [9]. Hemolysis, in turn, results in the release of the intracellular contents of RBCs. Lack of Hb compartmentalization leads to systemic nitric oxide scavenging and deteriorates endothelial function [10]. Also, Hb reacts with H_2_O_2_ (e.g., generated in inflammatory states), augments ferryl-Hb synthesis, and speeds up lipid peroxidation [11]. Hb or heme also activates Toll-like receptor 4 and supports proinflammatory signaling [12].

Consequently, in the current study, we aimed to assess the effect of melatonin on mouse blood redox systems in animals simultaneously exposed to ethanol and low-dose LPS. Such exposure closely mimics clinical situations in which alcohol intake is associated with increased intestinal permeability. Thus, the study was performed to (i) determine the effect of simultaneous ethanol-induced intoxication and inflammation caused by low-dose LPS on blood cell morphology, acid-induced and osmotic resistance of erythrocytes, and levels of glycated hemoglobin and oxidative stress biomarkers; (ii) identify the relationships between the oxidative stress biomarkers estimated by total antioxidant capacity, the concentration of diene conjugates and 2-thiobarbituric acid reactive substances (TBARS) as biomarkers of lipid peroxidation (LPO), and carbonyl derivatives of protein damage during acute ethanol-induced toxicity and a low-dose LPS exposure; (iii) clarify the hypothesis that melatonin treatment is an effective agent against destructive processes triggered by simultaneous action of alcohol and low-dose LPS.

## Materials and methods

### Animals and experimental design

The experiments were performed by the Guidelines of the European Union Council and the current laws in Ukraine and were approved by the Ethical Commission of the National State University in Chernihiv (2612/2016). Healthy male white Balb/c mice (*Mus musculus*), weighing about 20–30 g and aged about 2–3 months, were used in the experiments. The data were collected from 48 adult animals divided into eight groups.

### Experimental groups

Mice were randomly assigned into eight groups: (1) untreated control (6 animals), (2) melatonin treatment (6 animals), (3) acute ethanol-induced toxicity (6 animals), (4) LPS-induced inflammation (6 animals), (5) melatonin treatment + LPS-induced inflammation (6 animals), (6) LPS-induced inflammation + acute ethanol-induced toxicity (6 animals), (7) melatonin treatment + acute ethanol-induced toxicity (6 animals), and (8) melatonin treatment + LPS-induced inflammation + acute ethanol-induced toxicity (6 animals).

### Melatonin

The treatment of melatonin (Sigma-Aldrich Sp. z.o.o, Poznan, Poland) was delivered by intraperitoneal injection with 10 mg kg^−1^ of melatonin for 10 days. Melatonin was dissolved in a minimum volume of ethanol and diluted in 0.9% NaCl to yield a dose of 10 mg kg^−1^ body weight (b.w.), as described in previous studies [6]. Melatonin was intraperitoneally injected 30 min before ethanol and/or LPS exposure.

### Acute ethanol-induced toxicity

Acute exposure to ethanol was induced by intraperitoneal injection of ethanol in a dose of 0.75 g kg^−1^ b.w. per day. Ethanol was diluted from a 95% (v/v) solution to a concentration of 20% (v/v) with physiological saline (0.9%) and was administered as intraperitoneal (IP) injections at doses of 0.75 g kg^−1^ b.w. or in an injection volume 4.73 mL kg^−1^ b.w. per day during 10 days of the experiment as described by authors [13].

### Lipopolysaccharide

*E. coli* serotype 026:B6 (Sigma-Aldrich Sp. z.o.o, Poznan, Poland) injections of LPS were administered once, intraperitoneally, at a dose of 150 μg per mouse, as described by the authors [14, 15].

### Controls

Negative control mice were injected with 0.9% NaCl.

### Sampling and isolation of erythrocytes

Samples were collected at 24 h after the last drug administration and injection of Mel, ethanol, and/or LPS. Blood samples were taken from the caudal vein using syringes in less than 1 min and transferred to tubes with K_2_-EDTA. After centrifugation, plasma samples were removed and frozen at – 20 °C and stored until analysis.

### Acid-induced resistance of erythrocytes

Resistance was determined according to the method of the authors [16]. The method is based on the measurement of erythrocyte dynamic disintegration in 0.1N HCl, used as the hemolytic reagent. The acid resistance of erythrocytes is defined as the percentage of the disintegration of erythrocytes over time and was expressed as a curve.

### Osmotic resistance of erythrocytes

The Kamyshnikov method [17] was used to assay the osmotic resistance of erythrocytes. The method is based on the measurement of differences between the osmotic-induced hemolysis of erythrocytes in a mixture containing different concentrations of sodium chloride and urea. The absorbance of the mixture containing erythrocytes and 0.3M urea was defined as 100% hemolysis.

### Hematological profile

K_2_-EDTA blood was collected and analyzed automatically (Abacus Junior Vet, Diatron MI Zrt., Budapest, Hungary) to obtain the following data: red blood cell (RBC) count (10^6^/μL), white blood cell (WBC) count (10^3^/μL), lymphocyte (10^3^/μL), monocyte (10^3^/μL), neutrophil (10^3^/μL), lymphocyte (%), monocyte (%) and neutrophil (%), Hb (dl/g), packed cell volume (PCV), hematocrit (HCT) (%), mean corpuscular volume (MCV, fL), mean corpuscular hemoglobin (MCH, pg), mean corpuscular hemoglobin concentration (MCHC, g/dL), RBC distribution width (RDWc, %), platelet count (PLT, 10^3^/μL), packed cell volume (PCV, %), mean platelet volume (MPV, fL), and platelet distribution width (PDWc, %). Levels of glycated Hb in the blood of mice were estimated using the HemoCue HbA1c 501 (HemoCue AB, Angelholm, Sweden) system and was expressed as a percentage.

### Conjugated diene assay

The level of conjugated dienes was determined according to the Kamyshnikov method [17]. The structures of conjugated dienes with alternating double and single bonds between carbon atoms absorb wavelengths of 230–235 nm in the UV region and are expressed in nmol mL^−1^ of plasma.

### 2-Thiobarbituric acid reactive substance assay

TBARS were measured using the method described by Kamyshnikov [17]. TBARS level was expressed in nanomole of malonic dialdehyde (MDA) per milliliter of plasma.

### Assay of carbonyl derivatives of oxidatively modified proteins

OMP rate was estimated using the reaction of the resultant carbonyl derivatives of amino acids with 2.4-dinitrophenyl hydrazine (DNFH), as described by Levine et al. [18] and modified by Dubinina et al. [19]. Levels of carbonyl groups were determined spectrophotometrically at 370 nm (aldehydic derivatives, AD) and 430 nm (ketonic derivatives, KD), and expressed in nmol mL^−1^ of plasma.

### Superoxide dismutase activity assay

Superoxide dismutase (SOD, E.C. 1.15.1.1) activity was determined according to the Kostiuk et al. [20]. SOD activity was assessed according to its ability to dismutate superoxide produced during quercetin auto-oxidation in an alkaline medium (pH 10.0). Absorbance at 406 nm was measured immediately and after 20 min. Activity is expressed in units of SOD per milliliter of blood.

### Catalase activity assay

Catalase (CAT, E.C. 1.11.1.6) activity was determined by measuring the decrease of H_2_O_2_ in the reaction mixture by the Koroliuk et al. method [21]. One unit of CAT activity is defined as the amount of enzyme required for decomposition of 1 μmol H_2_O_2_ min^−1^ mL^−1^ of blood.

### Glutathione reductase activity assay

Glutathione reductase (GR, E.C. 1.6.4.2) activity was measured according to the method described by Glatzle et al. [22]. Enzymatic activity was assayed spectrophotometrically by measuring NADPH consumption. A blank without NADPH was used and the GR activity was expressed as nmol NADPH_2_ min^−1^mL^−1^ of blood.

### Glutathione peroxidase activity assay

Glutathione peroxidase (GPx, EC 1.11.1.9) activity was determined by the detection of non-enzymatic utilization of reduced glutathione (GSH) as the reacting substrate at 412 nm after incubation with 5,5-dithiobis-2-nitrobenzoic acid (DTNB) according to the Moin method [23]. GPx activity was expressed as nmol GSH min^−1^ mL^−1^ of blood.

### Ceruloplasmin level assay

Ceruloplasmin (CP, E.C. 1.16.3.1) level in the plasma was measured spectrophotometrically at 540 nm as described by Kamyshnikov [17]. Ceruloplasmin was expressed as milligrams per liter of plasma.

### Total antioxidant capacity assay

The TAC level in the plasma was estimated spectrophotometrically with Tween 80 oxidation at 532 nm by measuring the TBARS level following the method described by Galaktionova et al. [24]. The level of TAC in the sample (%) was calculated comparing with the absorbance of the blank.

### Statistical analysis

Results were expressed as mean ± S.D. All variables were tested for normal distribution using the Kolmogorov-Smirnov and Lilliefors tests (*p* > 0.05) and homogeneity of variance was checked by using the Levene’s test. The significance of differences in parameters between untreated control and treated groups was examined using a one-way analysis of variance (ANOVA). We also used Bonferonni’s post-test [25]. Statistical analysis was carried out 12 ways, i.e., the effect of melatonin, ethanol, and LPS was compared with those of the control group. The combined effect of melatonin and LPS, melatonin and ethanol, and LPS and melatonin was compared with the data of the melatonin-treated group, the LPS group, and the ethanol groups separately. The combined effect of the LPS, ethanol, and melatonin groups was compared with the data of the LPS and ethanol, LPS and melatonin, ethanol and melatonin groups separately. Differences were considered significant at *p* < 0.05. All statistical calculations were performed on separate data from each group with STATISTICA 8.0 software (StatSoft Inc., Poland).

## Results

The effects of the melatonin treatment, ethanol-induced toxicity, and endotoxemia caused by LPS and the combined effects of ethanol-induced toxicity and melatonin treatment, LPS and melatonin treatment, and LPS, ethanol, and melatonin treatment on blood morphology are presented in Table 1. ANOVA analysis of the melatonin-treated effects assessed in the 8 experimental groups revealed significant correlations among white blood cells (WBC) (*F*_7,41_ = 4.33, *p* = 0.001), lymphocytes (LYM; *F*_7,41_ = 4.38, *p* = 0.001), neutrophils (NEU; *F*_7,41_ = 3.40, *p* = 0.006), lymphocytes (LYM %; *F*_7,41_ = 2.44, *p* = 0.034), RBC (*F*_7,41_ = 2.26, *p* = 0.023), mean corpuscular volume (MCV; *F*_7, 41_ = 7.07, *p* = 0.000), red cell distribution width (RDW; *F*_7,41_ = 5.34, *p* = 0.000), mean platelet volume (MPV; *F*_7,41_ = 2.40, *p* = 0.037), and platelet distribution width (PDW; *F*_7,41_ = 4.88, *p* = 0.000).

**Table 1:** Effects of melatonin treatment on the blood morphological parameters at LPS-induced inflammation and acute ethanol-induced toxicity in mice (*n* = 6)

Blood morphology/groups	Untreated control	Ethanol	LPS	LPS + ethanol	Melatonin + LPS + ethanol
RBC, 10^6^/μL	7.91 ± 0.29	7.96 ± 0.17	6.40 ± 0.61	8.05 ± 0.14	7.96 ± 0.17
WBC, 10^3^/μL	4.79 ± 0.36	8.52 ± 1.36^**a**^ (*p* = 0.031)	3.56 ± 0.85	6.38 ± 0.31	5.53 ± 0.57^**ddd**^ (*p* = 0.035)
LYM, 10^3^/μL	3.60 ± 0.23	5.73 ± 0.86	2.64 ± 0.58	4.97 ± 0.19	4.10 ± 0.38
MON, 10^3^/μL	0.18 ± 0.04	0.28 ± 0.06	0.17 ± 0.09	0.22 ± 0.50	0.10 ± 0.02
NEU, 10^3^/μL	1.01 ± 2.26	2.52 ± 0.56^**a**^ (*p* = 0.037)	0.75 ± 0.20	1.20 ± 0.12	1.34 ± 0.19
LYM, %	75.71 ± 2.63	67.78 ± 1.18	72.42 ± 2.24	78.11 ± 1.77	74.50 ± 1.22
MON, %	3.70 ± 0.61	4.17 ± 1.15	3.78 ± 1.19	3.25 ± 0.65	1.70 ± 0.21
NEU, %	20.57 ± 2.26	28.08 ± 2.02	20.83 ± 2.22	18.60 ± 1.44	23.81 ± 1.12
Hb, g/dL	13.54 ± 0.59	13.67 ± 0.35	11.03 ± 1.28	13.71 ± 0.23	12.77 ± 0.47
HCT, %	42.70 ± 1.86	42.61 ± 0.81	35.52 ± 3.43	43.06 ± 0.59	41.36 ± 1.50
MCV, fL	53.71 ± 0.78	53.33 ± 0.21	55.50 ± 0.67	53.33 ± 0.33	57.16 ± 0.54^**cc**^ (*p* = 0.001)
MCH, pg	17.07 ± 0.20	17.15 ± 0.18	17.03 ± 0.53	17.07 ± 0.18	17.65 ± 0.24
MCHC, g/dL	31.74 ± 0.36	32.02 ± 0.37	30.7 ± 0.75	31.87 ± 0.38	30.88 ± 0.36
RDWc, %	18.13 ± 0.30	17.32 ± 0.10	18.52 ± 0.19	18.53 ± 0.16	19.20 ± 0.12^**c,cc**^ (*p* = 0.015) (*p* = 0.000)
PLT, 10^3^/μL	598.43 ± 39.53	474.0 ± 44.75	536.17 ± 38.32	636.33 ± 19.34	543.33 ± 52.88
PCT, %	0.47 ± 0.03	0.38 ± 0.04	0.41 ± 0.03	0.49 ± 0.12	0.41 ± 0.04
MPV, fL	7.81 ± 0.05	7.88 ± 0.1	7.57 ± 0.11	7.65 ± 0.08	7.43 ± 0.06
PDWc, %	30.91 ± 0.15	30.80 ± 0.32	29.52 ± 0.26^**b**^ (*p* = 0.002)	30.27 ± 0.17	29.71 ± 0.17

Results are expressed as mean ± S.D. Differences between experimental groups (*n* = 6) were analyzed by one-way ANOVA and Bonferroni post hoc tests. Differences were considered significant when *p* < 0.05

*Control*, untreated control animals; *LPS*, LPS-induced inflammation model; *Ethanol*, ethanol-induced toxicity model; *LPS + ethanol*, LPS-induced inflammation model + ethanol-induced toxicity model; *Mel + LPS + ethanol*, melatonin treatment at LPS-induced inflammation and ethanol-induced toxicity models

Significant differences between groups are designated as follows:

^a^Ethanol-induced toxicity group vs untreated control group

^b^LPS-induced inflammation group vs untreated control group

^c^Melatonin treatment + LPS-induced inflammation + ethanol-induced toxicity group vs LPS-induced inflammation group

^cc^Melatonin treatment + LPS-induced inflammation + ethanol-induced toxicity group vs ethanol-induced toxicity group

^ddd^Melatonin treatment + LPS-induced inflammation + ethanol-induced toxicity group group vs LPS-induced inflammation + ethanol-induced toxicity group

Melatonin did not cause statistically significant changes in blood morphological parameters. Acute ethanol-induced toxicity resulted in a significant increase in the WBC count, especially neutrophils. Melatonin treatment in ethanol-intoxicated animals normalized WBC counts to values similar to untreated control animals. A single dose of LPS caused the depletion of WBC, especially lymphocytes and neutrophils. Melatonin treatment in the LPS-exposed mice restored WBC counts compared with the LPS-exposed animals (WBC counts were similar to values measured in untreated control mice).

The combined effect of the acute ethanol-induced toxicity and LPS exposure resulted in a significant increase in counts of both RBCs and lymphocytes, compared with the LPS-exposed group. The neutrophil count and MCV values for this group were statistically significantly lower than in the LPS-only exposure group. The combined impact of LPS and ethanol statistically significantly decreased the neutrophil and PLT counts, compared with the ethanol-exposed group. Animals treated with LPS and ethanol had significantly increased RDWc values and PLT counts, compared with mice exposed with ethanol alone. Melatonin treatment in the LPS + ethanol group significantly reduced the WBC count and increased MCV values.

**Fig. 1:**
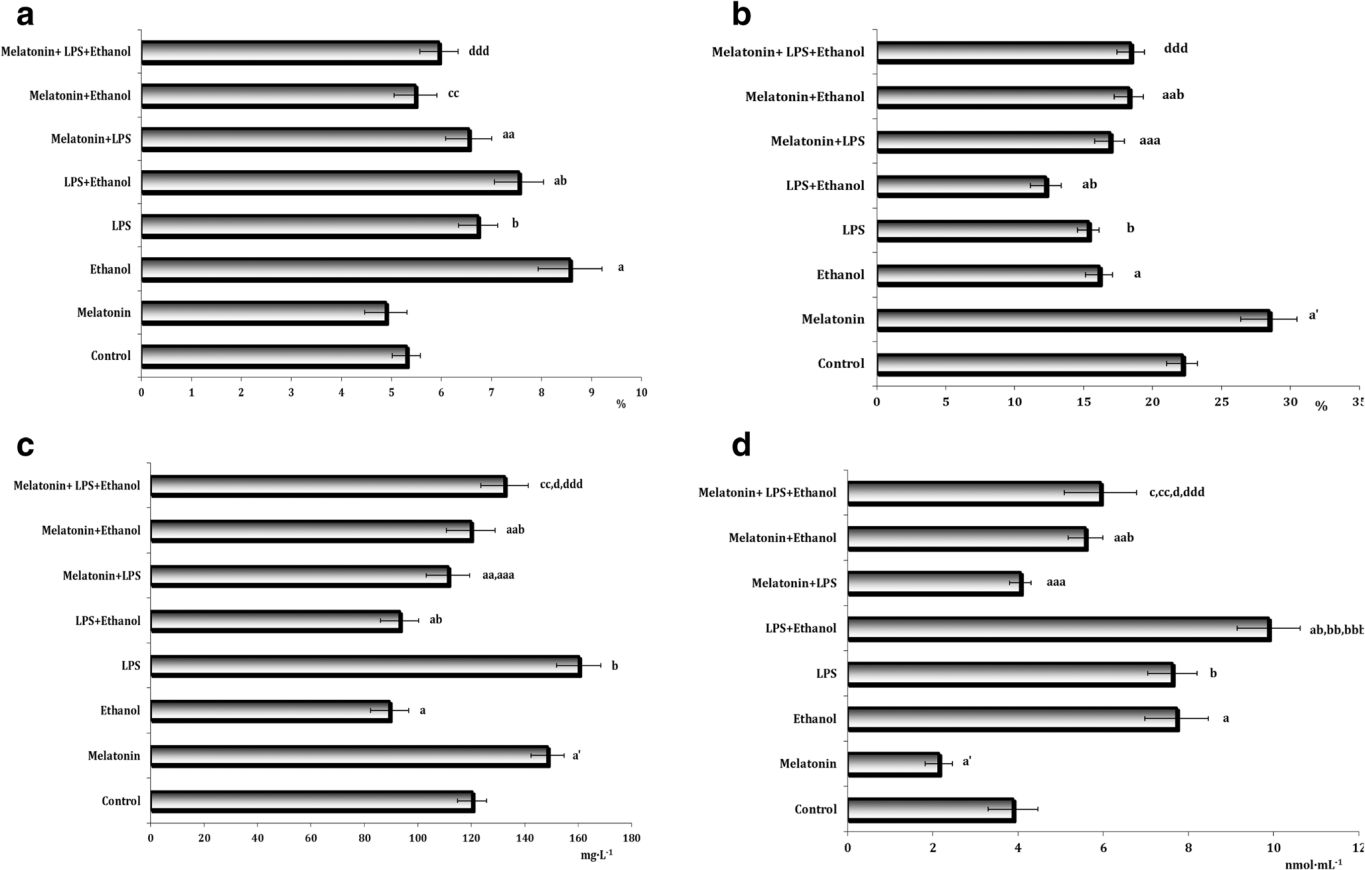
Effects of melatonin on levels of glycated hemoglobin (A, %), total antioxidant capacity (B, %), ceruloplasmin (C, mg L^−1^), and conjugated dienes (D, nmol mL^−1^) at LPS-induced inflammation model and acute ethanol-induced toxicity in mice. Results are expressed as mean ± S.D. Differences between experimental groups (*n* = 6) were analyzed by one-way ANOVA and Bonferroni post hoc tests. Differences were considered significant when *p* < 0.05. Control, untreated control animals; LPS, LPS-induced inflammation model; Ethanol, ethanol-induced toxicity model; LPS + Ethanol, LPS-induced inflammation model + ethanol-induced toxicity model; Mel + LPS + Ethanol, melatonin treatment at LPS-induced inflammation and ethanol-induced toxicity models. **a**—ethanol-induced toxicity group vs untreated control group (*p* < 0.05); **a’**—melatonin treatment group vs untreated control group (*p* < 0.05); **b**—LPS-induced inflammation group vs untreated control group (*p* < 0.05); **aa**—melatonin treatment at LPS-induced inflammation group vs LPS-induced inflammation group (*p* < 0.05); **aaa**—melatonin treatment at LPS-induced inflammation group vs melatonin treatment group (*p* < 0.05); **ab**—LPS-induced inflammation and ethanol-induced toxicity group vs untreated control group (*p* < 0.05); **bb**—LPS-induced inflammation and ethanol-induced toxicity group vs LPS-induced inflammation group (*p* < 0.05); **abb**—melatonin treatment at ethanol-induced toxicity group vs melatonin treatment group (*p* < 0.05); **bbb**—LPS-induced inflammation and ethanol-induced toxicity group vs ethanol-induced toxicity group (*p* < 0.05); **aab**—melatonin treatment at ethanol-induced toxicity group vs ethanol-induced toxicity group (*p* < 0.05); **c**—melatonin treatment at LPS-induced inflammation and ethanol-induced toxicity group vs LPS-induced inflammation group (*p* < 0.05); **cc**—melatonin treatment at LPS-induced inflammation and ethanol-induced toxicity group vs ethanol-induced toxicity group (*p* < 0.05); **ccc**—melatonin treatment at LPS-induced inflammation and ethanol-induced toxicity group vs melatonin treatment group (*p* < 0.05); **d**—melatonin treatment at LPS-induced inflammation and ethanol-induced toxicity group vs melatonin treatment at LPS-induced inflammation group (*p* < 0.05); **dd**—melatonin treatment at LPS-induced inflammation and ethanol-induced toxicity group vs melatonin treatment at ethanol-induced toxicity group (*p* < 0.05); **ddd—**melatonin treatment at LPS-induced inflammation and ethanol-induced toxicity group vs LPS-induced inflammation and ethanol-induced toxicity group (*p* < 0.05)

The concentration of HBA1c (Fig. 1A) differed significantly between the control and experimental groups (*F* = 8.96, *p* = 0.000). HBA1c levels were higher in the experimental groups, i.e., acute ethanol-induced stress group (*p* = 0.000), the LPS-induced inflammation group (*p* = 0.043), and combined LPS and ethanol group (*p* = 0.000), compared with their control counterparts. Melatonin treatment in LPS- and ethanol-exposed mice decreased HBA1c, compared with the ethanol and LPS groups.

We also estimated total antioxidant capacity (TAC), which can be used as a marker of the functional ability of the blood redox system to counteract oxidative stress (Fig. 1B). The TAC value was decreased statistically significantly after the ethanol, LPS, and combined ethanol and LPS exposure, compared with the untreated control mice. Melatonin treatment partially restored the blood redox system function (Fig. 1B).

Ceruloplasmin plays an important role in antioxidant defense. The concentration of ceruloplasmin was elevated in plasma after the melatonin treatment and LPS-exposed mice compared with values of untreated control mice (Fig. 1C). In contrast, both ethanol exposure alone, and combined with LPS exposure, resulted in reductions of ceruloplasmin concentration in plasma, compared with untreated control mice. Melatonin treatment of the LPS-exposed mice reduced the ceruloplasmin concentration in plasma compared with the mice exposed to LPS alone. In contrast, melatonin treatment in ethanol-exposed mice enhanced the ceruloplasmin value compared with the ethanol-only-exposed animals. Melatonin treatment in mice subjected to the combinative ethanol and LPS exposure increased the ceruloplasmin level in plasma compared with the group exposed simultaneously to ethanol and LPS (Fig. 1C).

Lipid reactions with ROS and subsequent transformations result in the formation of many low molecular weight degradation products. Conjugated dienes are the initial products of these processes (Fig. 1D). The exposure to the LPS and ethanol separately, as well as the combination of ethanol and LPS, was associated with elevated levels of free radical–induced oxidation of lipids in the initial stage of lipid peroxidation (LPO), compared with the untreated control group. After melatonin treatment, the initial substrate accumulation during LPO was lower than in the untreated control mice. Melatonin statistically significantly decreased the concentration of conjugated dienes in the ethanol, LPS, and ethanol + LPS groups.

**Fig. 2:**
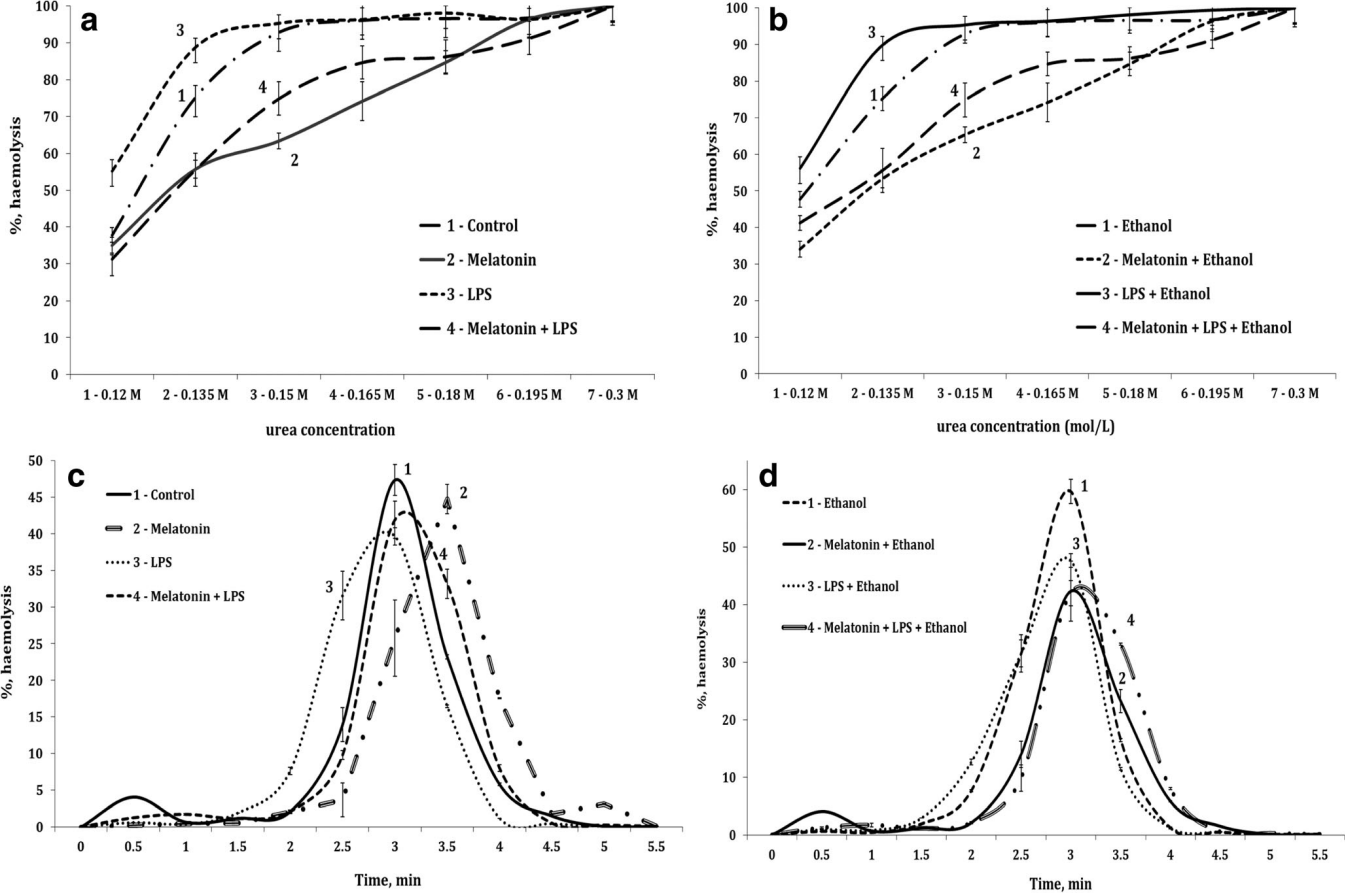
Osmotic resistance of erythrocytes (% of hemolyzed erythrocytes in solutions with increasing concentrations of urea) and acid-induced resistance of erythrocytes (% hemolyzed erythrocytes per minute) in the untreated control group, melatonin-treated group, LPS-exposed group, melatonin-treated + LPS group (A, C), acute ethanol-induced toxicity, melatonin treatment + ethanol-induced toxicity, LPS + ethanol-induced toxicity, melatonin treatment + LPS + ethanol-induced toxicity (B, D). Horizontal axis for Figs. 2A, B: solutions with different urea concentration (1–0.12, 2–0.135, 3–0.15, 4–0.165, 5–0.18, 6–0.195, 7–0.3 mol/L. Results are expressed as mean ± S.D

Ethanol and LPS exposure endangers RBC membranes. To identify changes of RBCs, we have determined the osmotic (Fig. 2A, B) and acid-induced resistance of erythrocytes to hemolytic agents (Fig. 2C, D). To elucidate patterns related to melatonin treatment, we divided all experimental series into two sectors. In Fig. 2, they are represented as parts A, B, C, and D. The dynamics of erythrocyte disintegration estimated by their resistance to hydrochloric acid (% hemolysis of erythrocytes per minute) increased after ethanol, LPS, and combined ethanol and LPS exposure, causing shifting of the curve to the left. Melatonin treatment keeps stable RBC membranes.

MDA is an end product of the terminal stages of LPO processes. The MDA concentration was significantly increased in the LPS-exposed mice compared with the untreated control group. Melatonin statistically decreased the MDA concentration in the group of combined ethanol and LPS exposure. It should be noted that the melatonin treatment alone caused a decrease in the MDA values in the plasma of mice compared with the untreated control group (Table 2).

**Table 2:** Effects of melatonin treatment on the plasma oxidative stress biomarkers estimated through levels of MDA, and aldehydic and ketonic derivatives of oxidatively modified proteins at LPS-induced inflammation and acute ethanol-induced toxicity in mice (*n* = 6)

Parameters/groups	Untreated control	Ethanol	LPS	LPS + ethanol	Melatonin + LPS + ethanol
MDA, nmol mL^−1^	16.23 ± 2.11	15.33 ± 2.57	23.14 ± 3.44^**b**^	33.16 ± 4.01^**c,d**^	21.63 ± 3.12^**e**^
Aldehydic derivatives of oxidatively modified proteins, nmol mL^−1^	4.22 ± 0.56	9.12 ± 2.33^**a**^	12.51 ± 1.16^**b**^	16.22 ± 2.33^**d**^	9.55 ± 2.11^**e**^
Ketonic derivatives of oxidatively modified proteins, nmol mL^−1^	3.98 ± 0.22	8.45 ± 1.96^**a**^	11.25 ± 1.13^**b**^	13.52 ± 1.75^**d**^	9.37 ± 1.63^**e**^

Results are expressed as mean ± S.D. Differences between experimental groups (*n* = 6) were analyzed by one-way ANOVA and Bonferroni post hoc tests. Differences were considered significant when *p* < 0.05

*Control*, untreated control animals; *LPS*, LPS-induced inflammation model; *Ethanol*, ethanol-induced toxicity model; *LPS + ethanol*, LPS-induced inflammation model + ethanol-induced toxicity model; *Mel + LPS + ethanol*, melatonin treatment at LPS-induced inflammation and ethanol-induced toxicity models

Significant differences between groups are designated as follows:

^a^Ethanol-induced toxicity group vs untreated control group

^b^LPS-induced inflammation group vs untreated control group

^c^LPS-induced inflammation + ethanol-induced toxicity group vs LPS-induced inflammation group

^d^LPS-induced inflammation + ethanol-induced toxicity group vs ethanol-induced toxicity group

^e^Melatonin treatment + LPS-induced inflammation + ethanol-induced toxicity group vs LPS-induced inflammation + ethanol-induced toxicity group

Intensification of free radical oxidation causes changes in proteins and their structure. Such changes are presented as carbonyl derivatives consisting of aldehydic (AD) and ketonic derivatives (KD). The concentration of carbonyl derivatives (AD and KD derivatives) was higher in the ethanol- and LPS-exposed groups compared with the untreated control mice. Melatonin treatment statistically significantly decreased concentration of AD and KD derivatives in the ethanol, LPS, and ethanol + LPS groups compared with the ethanol- and LPS-exposed mice (Table 2).

Activities of antioxidant enzymes in response to acute ethanol-induced toxicity, LPS-exposure, or the combination of these two models are presented in Table 3. Ethanol-induced toxicity increased SOD and GR, but reduced the GPx activity; endotoxemia caused by LPS exposure resulted in the elevation of the SOD, GR, and GPx activities in both groups compared with the untreated control group. The combined exposure to ethanol and LPS decreased the SOD, GR, and GPx activities compared with the ethanol group alone. Melatonin treatment decreased CAT and increased GPx activities compared with the untreated control group. Furthermore, melatonin treatment reversed the effects of LPS concerning SOD and GPx activities. Melatonin treatment in the acute ethanol-induced stress group increased CAT and decreased GPx activity compared with the ethanol-only group. After the combined ethanol and LPS exposure, melatonin treatment elevated GR activity compared with the ethanol- and LPS-exposed group.

**Table 3:** Effects of melatonin treatment on the activities of antioxidant enzymes at LPS-induced inflammation and acute ethanol-induced toxicity in mice (*n* = 6)

Groups/antioxidant enzymes	SOD, U mL^−1^	CAT, μmol H_2_O_2_·min^−1^ mL^−1^	GR, nmol NADPH_2_·min^−1^ mL^−1^	GPx, nmol GSH·min^−1^ mL^−1^
Untreated control	244.56 ± 45.29	12.22 ± 0.31	88.44 ± 16.22	56.22 ± 6.71
Ethanol	458.51 ± 30.52^**a**^	11.25 ± 1.12	125.47 ± 9.54^**a**^	49.78 ± 5.85
LPS	658.88 ± 58.64^**b**^	16.11 ± 1.04	156.55 ± 22.12^**b**^	118.43 ± 7.11^**b**^
LPS + ethanol	258.69 ± 34.66^**c,d**^	11.08 ± 2.89^**c**^	88.33 ± 9.41^**c,d**^	74.21 ± 16.29^**c,d**^
Melatonin + LPS+ ethanol	365.96 ± 56.17^**e**^	15.82 ± 1.95^**e**^	118.52 ± 7.69^**e**^	62.11 ± 9.87

Results are expressed as mean ± S.D. Differences between experimental groups (*n* = 6) were analyzed by one-way ANOVA and Bonferroni post hoc tests. Differences were considered significant when *p* < 0.05

*Control*, untreated control animals; *LPS*, LPS-induced inflammation model; *Ethanol*, ethanol-induced toxicity model; *LPS + ethanol*, LPS-induced inflammation model + ethanol-induced toxicity model; *Mel + LPS + ethanol*, melatonin treatment at LPS-induced inflammation and ethanol-induced toxicity models

Significant differences between groups are designated as follows:

^a^Ethanol-induced toxicity group vs untreated control group

^b^LPS-induced inflammation group vs untreated control group

^c^LPS-induced inflammation + ethanol-induced toxicity group vs LPS-induced inflammation group

^d^LPS-induced inflammation + ethanol-induced toxicity group vs ethanol-induced toxicity group

^e^Melatonin treatment + LPS-induced inflammation + ethanol-induced toxicity group vs LPS-induced inflammation + ethanol-induced toxicity group

## Discussion

The main findings of the current study are twofold: (1) melatonin exerts multilevel protection against oxidative stress in plasma through several mechanisms including stabilization of RBC membranes, augmented ceruloplasmin concentration, and blood antioxidant capacity; (2) melatonin limits lipid peroxidation and protein damage in plasma.

Gut-derived LPS plays a central role in the induction of steatosis, inflammation, and fibrosis associated with alcohol-induced liver diseases. It has recently been shown that suppression of oxidative and nitrative stress may restore proper gut-blood barrier function in rats exposed to alcohol [26]. To the best of our knowledge, we demonstrated for the first time that melatonin treatment may reinforce blood antioxidant redox systems in an animal model based on simultaneous acute exposure to ethanol and low-dose LPS.

The liver plays a central role in glucose and lipid metabolism, and consequently, alcohol-induced hepatic cell damage results in metabolic instability [6]. Treatment with melatonin reduces hepatic cell damage, steatosis, and the immigration of inflammatory cells; diminishes serum and tissue inflammatory cytokines levels, tissue lipid peroxidation, and neutrophil infiltration; and abolishes the apoptosis of hepatocytes in mice model of alcohol-induced liver injury [27], as well as an auxiliary treatment in patients with primary sepsis [28]. In turn, chronic low-grade inflammation results in a persistent imbalance between cellular energy availability and cellular and behavioral energy expenditure, and, as a consequence, the organism is exposed to constant instability concerning levels of glucose [29]. Melatonin may counteract metabolic disturbances through several mechanisms, such as regulation of insulin secretion and protection against pancreatic β cell exposure to oxidative stress [30]. Moreover, endogenous LPS concentration closely correlates with HbA1c [31]. Consequently, we observed a normalization of HbA1c in melatonin-treated groups, an observation that may also suggest an overall decrease of gut permeability in melatonin-treated animals.

Ceruloplasmin is a key antioxidant, which can inhibit oxidative stress produced through the action of ferrous ions. Ceruloplasmin competitively binds to Fe^2+^, oxidizes Fe^2+^ to Fe^3+^, and finally eliminates the products of this reaction [32]. Ceruloplasmin may also inhibit lipid peroxidation and effectively diminish the hydrolysis of RBC induced by Cu^2+^. We have previously demonstrated that melatonin can diminish ceruloplasmin concentration in animals suffering from low-intensity inflammation [7]. In the current study, we report a similar function of melatonin in response to ethanol and low-dose LPS exposure. Decreased ceruloplasmin concentration may suggest diminished oxidative stress and lower levels of exposure to Fe^2+^. Such reasoning is by the observed stabilization of RBC membranes by melatonin. Decreased RBC decomposition also limits plasma concentrations of Fe^2+^ [33] and, consequently, the need for increased ceruloplasmin activity.

It is clear that while both ethanol and LPS induce oxidative stress [6–8], the combination of both factors is more highly toxic to the organism. As a consequence, protein damage and lipid peroxidation in the plasma is highly exaggerated. Melatonin inhibits alcohol-induced increases in gut permeability in experimental animals [34]. We demonstrated that melatonin provides antioxidant protection also at the level of blood redox systems resulting in an effective decrease in conjugated dienes, malonic dialdehyde, and carbonyl derivatives in the animal model for alcohol intake combined with low-intensity inflammation.

To conclude, melatonin treatment in combination with acute ethanol-induced toxicity and low-dose LPS exposure decreased the WBC values and glycated hemoglobin concentrations, stabilized erythrocyte membranes, and limited lipid peroxidation and protein damage at initial and final stages through maintenance of the functional ability of the blood redox system to counteract pathological conditions. Thus, we unveiled a novel mechanism of melatonin protective action in alcohol-induced toxicity combined with low-intensity inflammation.
